# Regional variation in life history traits and plastic responses to temperature of the major malaria vector *Nyssorhynchus darlingi* in Brazil

**DOI:** 10.1038/s41598-019-41651-x

**Published:** 2019-03-29

**Authors:** V. M. Chu, M. A. M. Sallum, T. E. Moore, W. Lainhart, C. D. Schlichting, J. E. Conn

**Affiliations:** 1 Department of Biomedical Sciences, School of Public Health, University at Albany (State University of New York), Albany, NY USA; 2 Wadsworth Center, New York State Department of Health, Albany, NY USA; 3 Departamento de Epidemiologia, Faculdade de Saúde Pública, Universidade de São Paulo, São Paulo, SP Brazil; 4 Department of Ecology and Evolutionary Biology, University of Connecticut, Storrs, CT USA; 5 Present Address: Department of Pathology, University of Arizona College of Medicine, Tucson, AZ USA

## Abstract

The primary Brazilian malaria vector, *Nyssorhynchus darlingi* (formerly *Anopheles darlingi*), ranges from 0°S–23°S across three biomes (Amazonia, Cerrado, Mata Atlântica). Rising temperatures will increase mosquito developmental rates, and models predict future malaria transmission by *Ny*. *darlingi* in Brazil will shift southward. We reared F_1_ *Ny*. *darlingi* (progeny of field-collected females from 4 state populations across Brazil) at three temperatures (20, 24, 28 °C) and measured key life-history traits. Our results reveal geographic variation due to both genetic differences among localities and plastic responses to temperature differences. Temperature significantly altered all traits: faster larval development, shorter adult life and overall lifespan, and smaller body sizes were seen at 28 °C versus 20 °C. Low-latitude Amazonia mosquitoes had the fastest larval development at all temperatures, but at 28 °C, average development rate of high-latitude Mata Atlântica mosquitoes was accelerated and equivalent to low-latitude Amazonia. Body size of adult mosquitoes from the Mata Atlântica remained larger at all temperatures. We detected genetic variation in the plastic responses among mosquitoes from different localities, with implications for malaria transmission under climate change. Faster development combined with larger body size, without a tradeoff in adult longevity, suggests vectorial capacities of some Mata Atlântica populations may significantly increase under warming climates.

## Introduction

Although the average incidence of malaria decreased by 22% across the South American continent between 2010–2016, nearly all countries in the Amazonia biome experienced an increase, especially Venezuela in 2017^1,2^. In Brazil, Amazonia has long been a hotbed of malaria transmission^2–4^ and remains a complex region for the implementation of successful control interventions^5,6^. In particular, recent studies have highlighted the impact of deforestation^7^, high prevalence of asymptomatic cases, difficulties in surveillance and medical treatment, and human migration^8^ on the maintenance of malaria in this region.

An essential but under-investigated driver of malaria is the vector *Nyssorhynchus darlingi* (formerly known as *Anopheles darlingi*)^9^, distributed throughout much of Latin America. This species readily colonizes habitats with diverse ecological characteristics^10,11^, and in Brazil is widely distributed across three biomes, Amazonia, Cerrado and Mata Atlântica. There is evidence of genetic differentiation within and among *Ny*. *darlingi* populations across these biomes^12^, as well as within the Brazilian^13^ and Peruvian Amazonia biome^14^. Such differentiation suggests that a combination of genetic variation and phenotypic plasticity (the ability of individual genotypes to produce different phenotypes in different environments) may enable adaptation to a range of ecological conditions. Mosquito development can be affected by many environmental factors^15–18^ that directly and indirectly affect both vectorial capacity and malaria transmission^16–20^. Analyses of latitudinal clines often reveal that intraspecific trait variation is associated with environmental conditions^15,21^. Many insects (but not all^22^) such as the fruit fly (*Drosophila melanogaster*^21^), cabbage beetle (*Colaphellus bowringi*^23^), and mosquito (*Culex coronator*^24^) show increasing body size with increasing latitude. Such patterns of geographic variation can result from phenotypic plasticity, genetic adaptation, or a combination of the two^25^.

Phenotypic plasticity is a property of the genotype, and reaction norms can be studied to identify environmental, genetic, and genotype-by-environment effects^26,27^. Plasticity has been shown to influence the variability in host preference^28^ and ability to colonize new ecological niches of the African malaria vector *Anopheles gambiae s*.*s*.^29^. However, the study of phenotypic plasticity in life history traits of *Ny*. *darlingi* has been limited to wing size and shape^30,31^ and gonotrophic concordance^32^. In contrast to field collections, data from laboratory-reared populations can be used to disentangle genetic and plastic effects^33^.

Temperature is well documented to influence body size and development time in insects^34^, and all life stages of mosquito populations^35–37^. Temperatures are projected to rise 1–4 °C over the next 100 years as a result of climate change^38^. Changes in temperature can influence malaria distribution because female biting rate, adult mortality rate, parasite development rate, vector competence and, accordingly, vectorial capacity are temperature-dependent parameters^17^. Currently, malaria transmission has been recorded at average monthly temperatures up to 32 °C in Brazil^39^. Using data from *An*. *gambiae*, *An. stephensi* and *An. pseudopunctipennis*, models predict that mosquito populations can remain stable up to 33 °C^40^. In contrast, models and experiments found that the optimal temperature for *Plasmodium spp*. is around 25 °C^17,41^.

**Figure 1:**
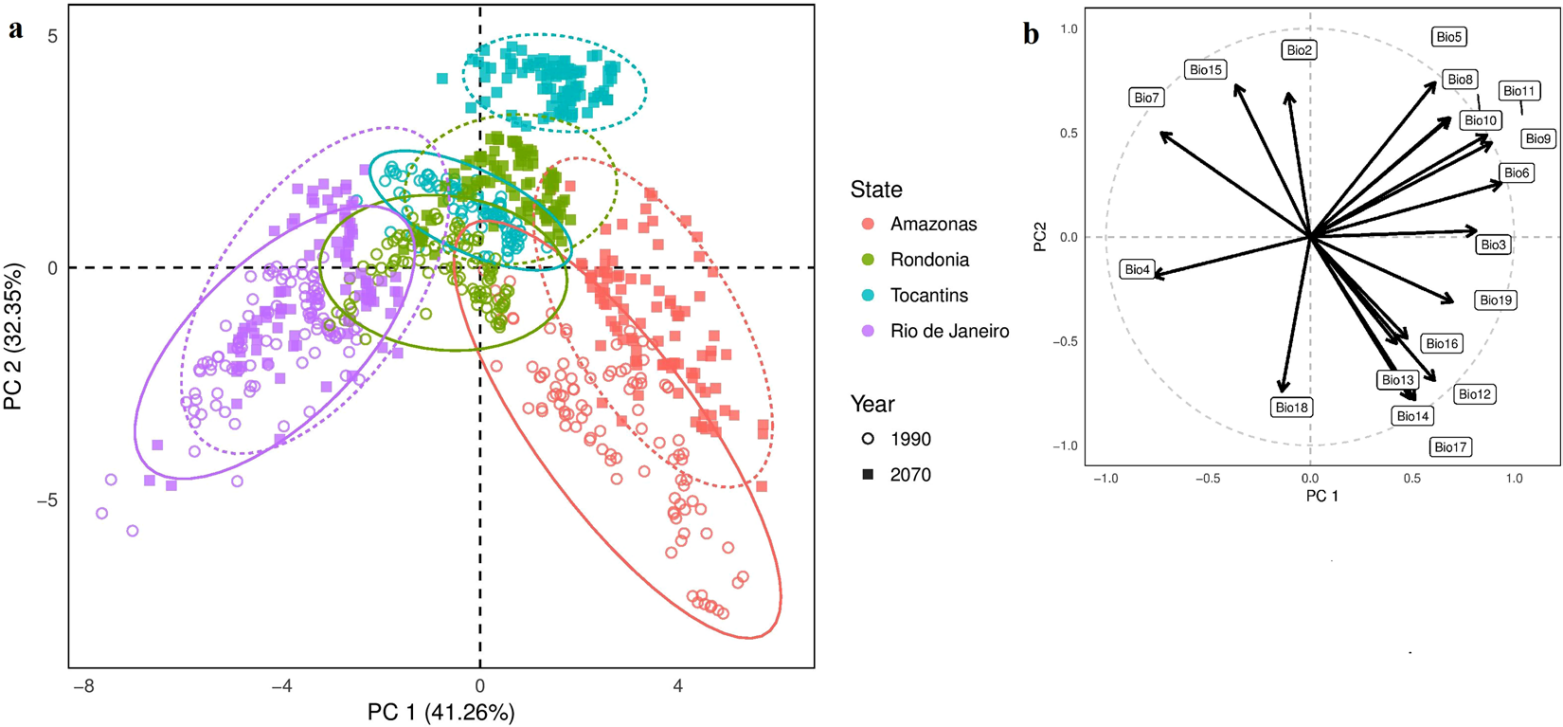
PCA of past (1960–1990) and future (2070) climates in each state (**a**) extracted based on 100 random GPS localities within each state and all 19 Bioclim variables at 30 arc second resolution, (**b**) correlation plot of 19 bioclimatic variables. Both past (1960–1990) and future (2070) climate data are from WorldClim^82^. The states of Amazonas and Rio de Janeiro have the most disparate climates now and in the future. Rondônia has little overlap with Amazonas despite being in the same biome. Rondônia and Tocantins, states at the same latitude but in different biomes, overlap some in current climate, but not in the future. In an extreme case, Tocantins shows no overlap between current and future climates. See additional details in supplement and Supplementary Table S5 for variable descriptions.

It is unclear how future climate change will affect the future distribution of malaria, as such change impacts every component of transmission, from vector dynamics to parasite development rate^37,42,43^. Current *Ny*. *darlingi* distributions and climate estimates have been used for projection models, and these forecast range expansion of this vector and increased intensity of malaria in southern Brazil^10,11^, due to the shifts in climate expected for Brazil (Fig. 1).

Life history trait estimates can be useful in directing control strategies^44,45^ and malaria elimination will require combinations of interventions at different life stages^46,47^ with frequent monitoring and evaluation^48^. In general, mosquito vector research has focused on adult components, often overlooking features of larval development important for the assessment of vectorial capacity. The latter integrates different aspects of mosquito and parasite life histories, e.g., daily female survival rate, human blood feeding rate, vector density, and the extrinsic incubation period^19^. Larger mosquitoes with increased survival and fecundity^49,50^ have been associated with increased food quantity^16,18,51^, reduced larval density and competition^16^, and lower rearing temperatures^15,17,51,52^. Laboratory experiments using colonies of *An*. *gambiae* and *An*. *stephensi* determined that increasing mean ambient and diurnal temperature reduced overall vectorial capacity through a reduction in parasite viability and mosquito survival rate at temperatures exceeding 30 °C^20^. Recent modeling^40^, field studies^53^ and laboratory rearing experiments^16,18,20^ measured life history traits under a range of environmental conditions and found that vectorial capacity values were highly variable. This suggests possible roles for genetic differentiation or phenotypic plasticity of vectors affecting rates of disease transmission.

**Figure 2:**
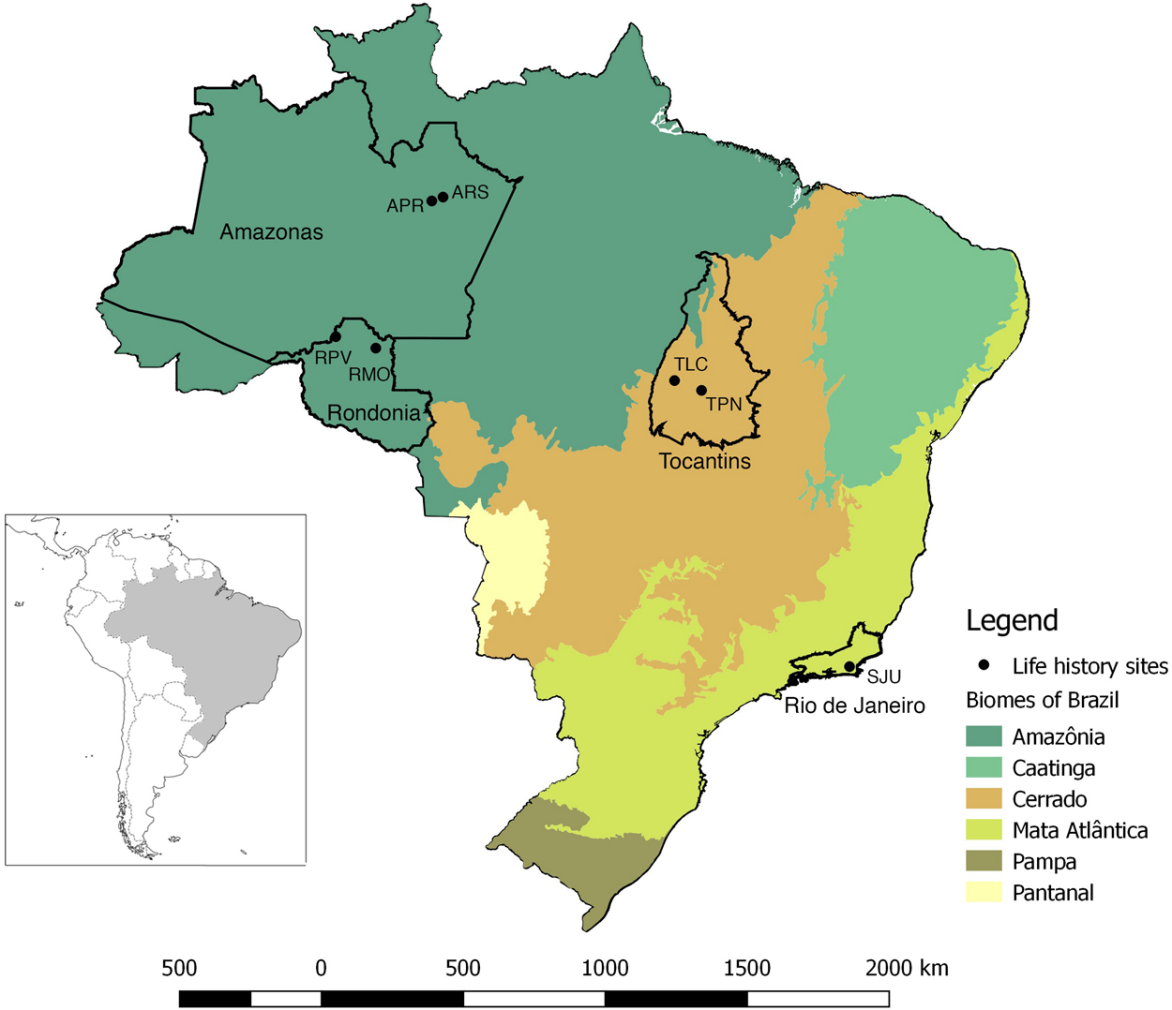
Map of *Ny*. *darlingi* collection sites across Brazil. Biome distributions are from publicly available maps^83^.

Theoretical models have shown that populations with high phenotypic and genetic variability are expected to have broader niches, increased population growth rate, and increased resilience to environmental change^54^. Comparative study of phenotypic plasticity of life history traits can provide significant insight into the ecological genetics of widely distributed species. In the present study, we investigate how differences in rearing temperature and population affect life history traits of *Ny*. *darlingi* from three Brazilian biomes. Our experimental design allows us to partition phenotypic variation among populations into plastic and genetic components as well as their interaction (i.e., whether there is genetic variation for plasticity). Studying populations from different Brazilian states across the distribution of *Ny*. *darlingi* (Fig. 2) under a range of temperature treatments will provide baseline estimates of genetic variation and phenotypic plasticity that can inform future modeling efforts, tailor vector control efforts, and guide the design of novel anti-malarial strategies^42^.

## Results

### Laboratory rearing and survivorship

The initial dataset consisted of 3,430 individuals; 778 died before adult emergence. Proportional tests were conducted, and the interaction of temperature and state marginally affected larval survival to adulthood (M^2^ = 10.9, 6 *d*.*f*., *p* = 0.09) (Supplementary Table S1). In general, survivorship to adulthood in the laboratory decreased with increasing latitude and temperature, but the significant interaction of temperature and state indicates that the magnitude of temperature-related reduction is dependent on state (Supplementary Tables S2–S3). Except for Amazonas state, mosquitoes from all states had significantly higher mortality at 28 °C: populations of mosquitoes from Rondônia and Tocantins had ~16% reduced survival, whereas for Rio de Janeiro, a much steeper reduction of nearly 60% was observed (Supplementary Fig. S1 and Table S4). All further analyses were conducted on the 2,652 individuals that survived to adulthood (Table 1).

**Table 1:** Characteristics of seven collection sites in Brazil and sample size of *Ny*. *darlingi* tested in laboratory for life history traits at each of three temperatures (total n = 2,652).

Biome	State	Locality	Latitude	Average mean temp (min, max)*	No. mosquitos at each temp.		
					20 °C	24 °C	28 °C
Amazon	Amazonas	ARS	−2.864	27.6 (26.9, 28.6)^a^	134	135	130
		APR	−3.028	27.7 (26.7, 29.2)^a^	198	198	179
	Rondônia	RPV	−8.742	25.7 (23.2, 26.6)^b^	181	184	141
		RMO	−9.223	25.6 (22.8, 26.5)^b^	192	197	160
Cerrado	Tocantins	TLC	−10.7	28.4 (23.3, 35.4)^a^	92	84	71
		TPN	−10.796	27.5 (26.2, 28.6)^a^	32	35	24
Mata Atlantica	Rio de Janeiro	SJU	−22.611	21.2 (20.5, 22.3)^c^	129	123	33

*Temperature data are means and ranges of annual mean temperatures (2010–2016) for local weather stations^84,85^. Letters to the right of average mean temperatures are derived from Tukey HSD after ANOVA and indicate significant differences in temperatures among Localities (p-value < 0.01).

### Variation in life history due to temperature and state

**Figure 3:**
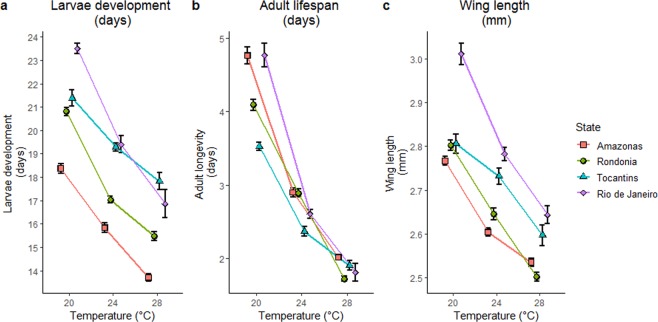
Average duration of larvae development (days, **a**) average adult life (days, **b**) and wing length (mm, **c**) with standard error bars graphed by state. See Supplementary Fig. S4 for significance of differences among states.

Analysis of Variance (ANOVA) showed significant variation in adult longevity, larval development and wing length due to temperature treatment and state of origin (Table 2). Variation in traits *among* states *within* temperature treatments is evidence of significant additive genetic variation among states (Figs 3 and S4). Significant differences *within* states *across* temperature treatments is evidence of phenotypic plasticity (Figs 3 and S4). The significant interaction between temperature and state (Table 2) indicates that *Ny*. *darlingi* populations from these regions also have genetic differences in plastic responses of larval development, adult longevity and wing length (i.e., differences in slopes in Fig. 3).

**Table 2:** ANOVA of life history traits by state and temperature.

Trait	Source	*d*.*f*.	MS	F	*p*
Larvae development	State	2	2349.67	530.91	**<2.2e-16**
	Temperature	3	444.26	100.38	**<2.2e-16**
	S × T	6	38.1	8.61	**4.16e-08**
Adult longevity	State	2	505.15	883.66	**<2.2e-16**
	Temperature	3	11.44	20.01	**6.66e-10**
	S × T	6	9.53	16.67	**4.44e-15**
Wing length	State	2	6.4327	459.61	**<2.2e-16**
	Temperature	3	0.4964	35.46	**9.77e-15**
	S × T	6	0.1388	9.92	**2.52e-09**

Significant *p*-values are in bold, *d*.*f*. - degrees of freedom; MS - mean squares.

Increasing temperature significantly reduced the average length of larval development time within each state (Figs 3a and S4). At the latitude extremes, Amazonas (Amazon) and Rio de Janeiro (Mata Atlântica) larval development differed by 4 to 6 days at all temperatures (Supplementary Fig. S4). Average larval development was distinct by latitude in the low temperature treatment (20 °C), but this distinction was lost in the higher temperature treatments (Supplementary Fig. S4). Within Amazonia, larval development of populations from Amazonas and Rondônia states differed by 2 or 3 days at all temperatures (Fig. 3a and Supplementary Table S6). Duration of pupal stage (Supplementary Fig. S2 and Table S7) and total time to emergence (larval and pupal stages) (Supplementary Fig. S3 and Table S8) showed similar trends.

Average unfed adult life span was significantly reduced as temperature increased within each state (Figs 3b and S4). At the latitude extremes, Amazonas (Amazon) and Rio de Janeiro (Mata Atlântica) had equivalent adult life spans at both 20 and 28 °C (Supplementary Fig. S4). Within Amazonia, adult life spans of populations from Amazonas and Rondônia states differed only at 20 °C and 28 °C (Fig. 3b and Supplementary Table S9).

Average wing length was significantly reduced with increasing temperature within each state (Fig. 3c, Table 2, Supplementary Fig. S4 and Table S10). Amazonia (Amazonas, Rondônia) populations and the Mata Atlântica (Rio de Janeiro) population were distinct at all temperatures, with average differences between 0.1 and 0.2 mm (Supplementary Fig. S4).

### Wing length increases with latitude in field and lab populations

**Figure 4:**
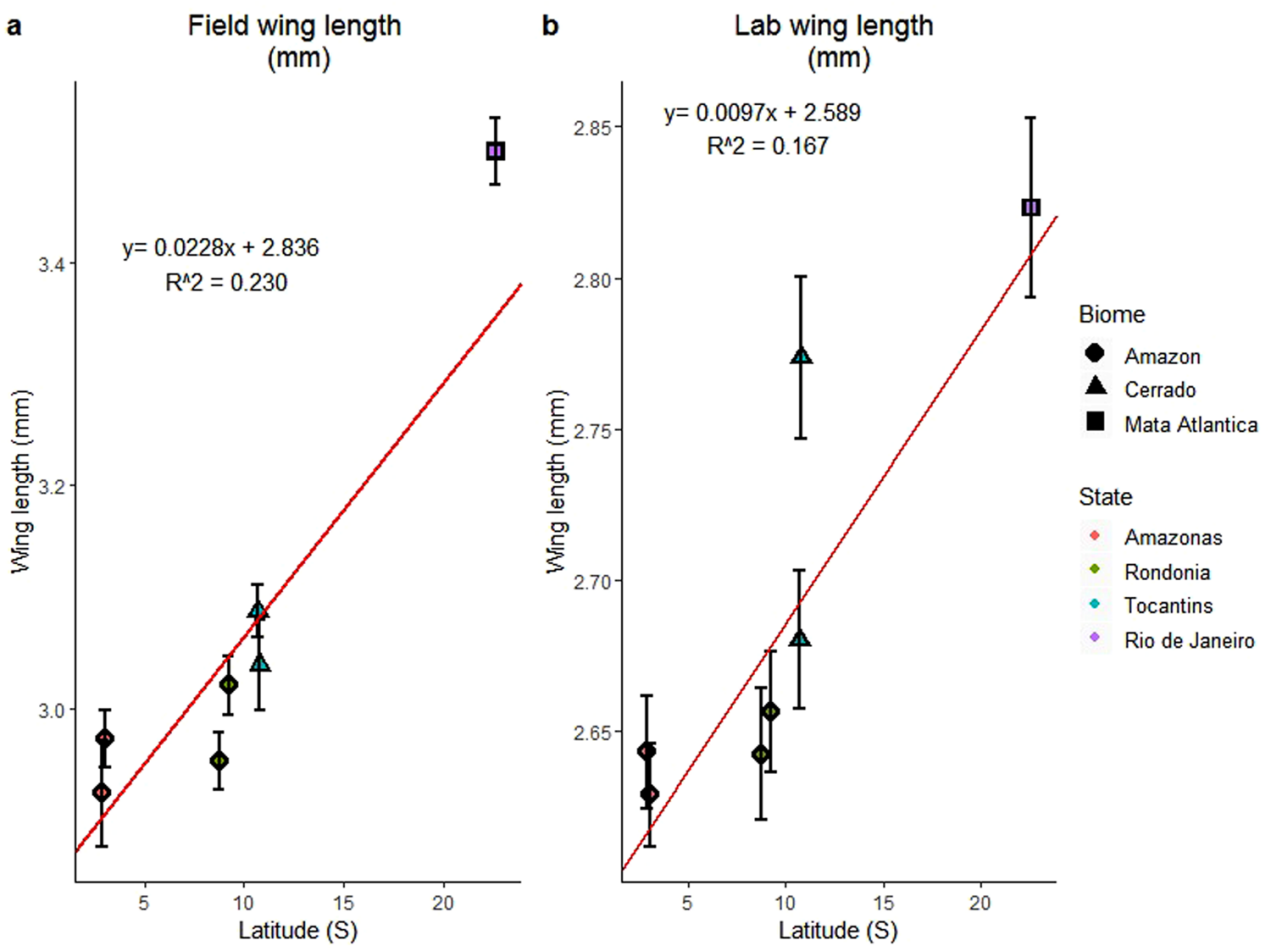
Evidence for Bergmann’s Rule. (**a**) Means (±SE) of population wing length measurements of field-collected mosquitoes are positively associated with latitude. (**b**) Means (±SE) of populations across temperature treatments for lab-reared, adult mosquitoes associated with latitude of origin, showing evidence of a genetic basis for Bergman’s cline in *Ny*. *darlingi*. Regression lines and equations correspond to OLS analysis. Note: scales of (**a**) and (**b**) y-axes differ.

Field-collected mosquitoes showed a Bergmann cline, where wing lengths increased with increasing latitude, and Rio de Janeiro state mosquitoes were the largest (Fig. 4a). We also find evidence of a genetic basis for the cline, with lab-reared mosquitoes also showing a positive relationship between wing lengths and latitude of origin (Fig. 4b). There was a greater difference in size of F_1_ lab-reared adults between the Tocantins state sites compared to the field-collected females from those sites (Fig. 4).

### Total lifespan decreases with increasing temperature

**Figure 5:**
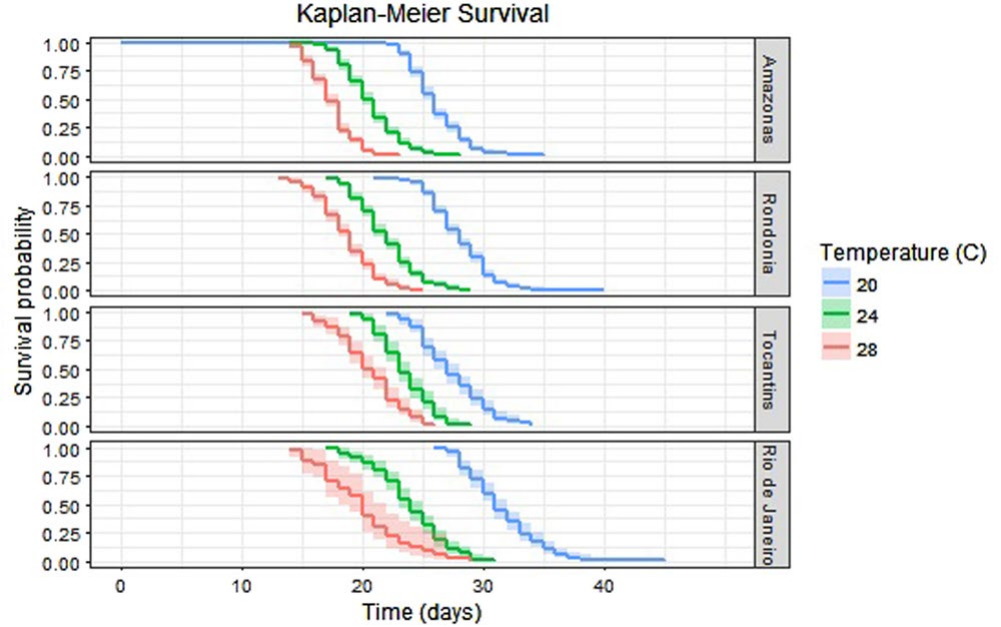
Kaplan Meier survival curves of mosquitoes that eclosed to adult (n = 2,652). These visualize total lifespan probabilities of mosquitoes within each state at each temperature treatment over time. There is a significant interaction between state and temperature, i.e., there are significant differences in the shapes of the survival curves.

The Kaplan-Meier survival curves indicated decreased lifespan within and across states as temperature increases (Fig. 5 and Table 3). Cox regression showed that increasing temperature within populations (*z* = 26.6, *p* < 2e-16) led to a significant decrease in survival, while a significant interaction of temperature with increasing latitude (*z* = 3.7, *p* < 2e-4) indicated that the 4 states differ in their overall survivorship curves.

**Table 3:** Probability of survival (±standard error) of the 2,652 mosquitoes that eclosed at 4 time points (15, 20, 25, 30 days post hatch) by temperature and state.

		20 °C	24 °C	28 °C	20 °C	24 °C	28 °C
		Amazonas state			Rondônia state		
Probability survival (±standard error)	Day 15	0.99 ± 0.003	0.86 ± 0.019	0.49 ± 0.028	1.0 ± 0.00	0.95 ± 0.011	0.70 ± 0.026
	Day 20	0.59 ± 0.026	0.09 ± 0.016	0	0.94 ± 0.011	0.25 ± 0.022	0.063 ± 0.014
	Day 25	0.03 ± 0.009	0	0	0.23 ± 0.022	0.008 ± 0.004	0
	Day 30	0	0	0	0.005 ± 0.004	0	0
		**Tocantins state**			**Rio de Janeiro state**		
Probability survival (±standard error)	Day 15	1.0 ± 0.00	1.0 ± 0.00	0.88 ± 0.033	1.0 ± 0.00	0.98 ± 0.011	0.69 ± 0.08
	Day 20	0.92 ± 0.024	0.59 ± 0.045	0.25 ± 0.045	0.99 ± 0.007	0.63 ± 0.044	0.21 ± 0.071
	Day 25	0.28 ± 0.04	0.017 ± 0.012	0	0.69 ± 0.041	0.07 ± 0.024	0.03 ± 0.029
	Day 30	0	0	0	0.05 ± 0.019	0	0

Higher probabilities at later days indicate longer preadult lifespans (Figs S2 and S3).

## Discussion

Most laboratory rearing studies of Anophelinae malaria vectors have relied on specimens from established colonies^16,18,37,50,55–58^, and thus, the range of biomes and latitudes of the populations in our study had not previously been examined^29,36^. Although studies using colony-reared specimens are invaluable, studying the F_1_ generation of wild-captured adult females has provided us with new insights into regional differences, including differences in both the mean values of traits and their plasticities.

Our findings indicate that geographic variation in *Ny*. *darlingi* life history traits results from both genetic differences among localities as well as plastic responses to differences in temperature. Mosquitoes from higher latitudes are genetically distinct (larger with longer lives) at 20 °C, but these differences in size and adult lifespan are diminished as temperatures rise to 24° and 28 °C. Our laboratory results verify that the wing size-latitude cline seen for field-collected populations is genetically based (Fig. 4). Genetic variation in life history traits indicates that *Ny*. *darlingi* could adapt to novel environmental conditions and may at least partly explain its success as the predominant South American malaria vector, colonizing many anthropogenic habitat types^59,60^. Further, our results demonstrate that there is genetic variation in the plastic responses among mosquito populations derived from different localities: the reaction norms (shapes of the curves) depicting responses to temperature vary among populations of different regions (Fig. 3).

Larval rearing studies have previously shown how rearing conditions, such as reduced nutrition and increased competition, reduced adult fitness^16,57,61^. In agreement with other mosquito life history studies^16,56,62^, increasing temperature reduced the average duration of larval development, adult life span, body size, and overall survival. The lowest latitude population in Amazonas state had the fastest larval development at every temperature. Mosquitoes from the population with the coolest climate, Rio de Janeiro state, developed nearly 5 days slower at the lowest temperature tested (20 °C) than those from Amazonas. However, the average unfed adult lifespan of *Ny*. *darlingi* from these two states was the same (~5 days). A study of colony *An*. *gambiae* found that longer development at a lower temperature resulted in larger mosquitoes, but adult longevity was the same across three larvae rearing temperatures^61^. We also found a positive association between development time and body size, but, in contrast, we found large differences in adult longevity across temperatures (Fig. 3b).

Research on conditions, such as temperature, that affect vector development has become a priority recently, because of the impact on malaria-relevant traits, e.g., vector competence^63^ or insecticide resistance^55^. Here, we show that, as expected, larval development, adult size and lifespan of *Ny*. *darlingi* are significantly affected by temperature. Most strikingly, we demonstrated that the nature of the plastic responses to temperature varies among regions: populations from warmer climates have flatter reaction norms in response to temperature, especially in relation to larval development (Fig. 3a). We found that difference in larval development (Fig. 3a) and adult size (Fig. 3c) are maintained at higher temperatures. Taken together, these results suggest that significant population-level differences in vectorial capacity will emerge as climate continues to warm and malaria moves south.

However, we also need to consider the significant mortality experienced by high-latitude mosquitoes. We are unsure why mosquitoes from Tocantins and Rio de Janeiro states should have lower overall survival in the laboratory – perhaps due to differences in their ability to tolerate laboratory humidity or constant temperature. Even if we set their overall survival rates aside, there is still a marked increase in mortality in these populations at 28 °C. Thus, reduced survival at higher temperatures may offset the life history advantages noted above that surviving individuals from these populations have gained. Of note is the broad spread of survival values among families within the Rio de Janeiro population - to the extent that this variation reflects genetic differences, the Rio de Janeiro population may still contain significant raw material for adaptation to higher temperatures.

### Possible study limitations

Our findings provide baseline information on the general relationship of temperature to larval development and adult traits, though there are some limitations. First, our study of F_1_ progeny could not exclude the impact of maternal effects, such as maternal fitness or nutritional content of blood meal prior to egg laying. There is no reason to believe, however, that such effects would be biased to be stronger in some localities than others. Second, there has been an increase in deforestation^7^ and urban development within the biomes that has altered the landscape at a local level, obscuring biome-level comparisons to some degree by introducing greater environment heterogeneity. Additionally, diurnally fluctuating temperatures and feeding of adults may produce results that are more useful for prediction of malaria transmission. In a previous study, fluctuating temperature, compared to constant temperatures, increased the rate of parasite development at lower temperatures, with the opposite effect observed at higher temperatures^64^. Meanwhile, models investigating temperature fluctuations on mosquito population dynamics found that total adult abundance might be reduced compared to constant temperatures^65^.

All localities were sampled just once in 2016 during lower precipitation months (Supplementary Table S1). The adult longevity observed in our study deviates from natural adult longevity that would involve regular adult feeding. For example, in *An*. *gambiae*, a colony-sourced population that was sugar-fed lived an average of 22 days^66^ compared with a field-sourced population that lived an average of 2 days when provided only water as adults^67^. Such differences between lab vs. field adult longevity preclude us from using lab longevity for vectorial capacity calculations^16^.

Recent colony establishment of *Ny*. *darlingi* will facilitate additional studies on mosquito development, with research already underway on vector competence^68^. Future laboratory work should be continued on field populations to investigate regional variation of blood feeding, fecundity, and vectorial capacity. Characterization of field populations will ultimately provide local information that can be updated to monitor the changing vector landscape and the effectiveness of disease abatement strategies.

## Conclusion

Projection models suggest that, in addition to the expected temperature increases^38^, climate differences among regions will become more pronounced in the future (Fig. 1). Our results indicate that higher latitude populations of *Ny*. *darlingi* may be able to tolerate a larger range of temperatures than they currently experience. This supports models suggesting that climate change is expected to increase the intensity of malaria cases in South America with increased abundance of *Ny*. *darlingi* in the south^10^.

Populations of *Ny*. *darlingi* vary not just for life history trait means, but also for how those traits respond to temperature variation. Importantly, this suggests that the relatively slow development at lower temperatures of higher latitude populations (such as those in the Mata Atlântica), may become much more rapid as temperatures increase. Adult longevities are not different among populations at high temperatures (Fig. 3b), but higher latitude populations stay larger at high temperatures.

The combination of faster development, and larger body size, without a tradeoff in adult longevity could lead to higher vectorial capacities of these southern populations. While the Rio de Janeiro population, as a whole, has much lower survival to adulthood in the lab at 28 °C, some families have much higher survival. If lower survival is not a factor in the field, or if the variation for survival in this population has a genetic basis, our results suggest that the Rio de Janeiro population may adapt to higher temperatures. Coupled with its life history characteristics at high temperatures, this could lead to significant increases in vectorial capacity in warming climates.

## Methods

### Mosquito collection

Mosquitoes were collected across four states representing 3 biomes: Amazonas, Rondônia (Amazonia biome), Tocantins (Cerrado biome) and Rio de Janeiro (Mata Atlântica biome) (Fig. 2). Collection-site pairs within a state were chosen using the following criteria: (i) proximity to forest edge and larval habitat suitable for *Ny*. *darlingi*; (ii) proximity to human dwellings; (iii) same latitude (±1°) and between 100–200 km apart; and (iv) no significant geographic barrier (river, mountains) between within-state pairs. However, for Rio de Janeiro, only one site yielded mosquitoes; repeated collection attempts failed at a second site where *Ny*. *darlingi* was known to occur^12^. Mosquitoes were collected in the evening for 5 hours (17:00–22:00) using barrier screens as described in Moreno *et al*.^69^. Blood-fed female mosquitoes from barrier screens were morphologically identified as *Ny*. *darlingi*^70^ and maintained individually in a humid box and provided *ad libitum* sucrose solution during transport to the laboratory (Laboratório de Entomologia da Faculdade de Saúde Pública, Universidade de São Paulo). Collection and egg laying data are provided (Supplementary Table S11).

### Biome descriptions

The Amazonia biome comprises ~61% of Brazil and is characterized by year-round high temperatures, high humidity, and a complex forest-river ecosystem. The Cerrado covers ~21% of Brazil, stretching across the central, semi-humid grasslands with perennial warm temperatures. The somewhat fragmented, mostly coastal, Mata Atlântica supports a cooler, humid climate and comprises mainly dense second growth forests with multilevel canopies (Fig. 2)^3,60,71,72^.

### Egg oviposition

Mosquitoes were allowed sufficient time for digestion and egg development before individual preparation for egg laying, either within 24 hours of arriving at the laboratory or 36–48 hours post blood meal. Each mosquito was briefly anesthetized with ethyl acetate vapor, the left wing removed, and then each mosquito was placed in an individually labeled oviposition cup containing distilled water for 36–48 hrs. at room temperature (26 °C ±2). Fine mesh was laid on top of the egg cups to minimize disturbance and prevent individual mosquitoes from escaping or disturbing other egg cups. Larvae from each cup constituted an individual maternal family and were not pooled with other larvae, and populations were randomized to minimize potential maternal effects.

### Mosquito rearing

All larvae, pupae and adults were maintained in temperature and light controlled chambers (12:12 light:dark cycle). Environmental chambers were set to 20 °C, 24 °C, 28 °C ± 1 °C. The temperatures used were selected to reflect the natural temperature range of *Ny*. *darlingi* across sites^73^ and to avoid laboratory rearing temperature extremes^52^. Data loggers (iButtons; iButton Link, LLC, Whitewater, WI, USA) were placed in all chambers to record and monitor temperature hourly to ensure a constant environment.

Larvae hatched within 1–2 days of oviposition. Upon hatching, the first 45 larvae obtained per egg cup were reared, 15 at each of three different temperatures (20 °C, 24 °C, 28 °C). Larvae were randomly assigned to temperatures for rearing in groups of 5. Larvae were reared in 6-well plates, each well containing 3 mL deionized water and 5 larvae, and fed 2 mg of food daily comprising a finely ground mixture (by weight) of Tetramin Tropical flakes (28%), Bettamin Tropical flakes (28%), Tetraveggie Spirulina (27%), Bettamin pellets (16.95%) and bee pollen (0.05%). Any larval deaths were recorded at the same time daily. Larvae were transferred to a clean 6-well plate with fresh water every other day after the 4^th^ day post-hatching. Pupae were collected daily and transferred into individual rearing vials containing 3 mL of deionized water and allowed to eclose. After emergence, adult mosquitoes were transferred to individual rearing vials containing a moistened cotton ball to provide an environment that was consistently between 70–80% relative humidity. Vials were capped with a water-moistened cotton ball to provide additional water; no food was provided. Mortality of pupae and adults was recorded daily.

### Wing measurements for body size estimation

The left wing, collected at either egg laying (field) or natural death (lab reared), was mounted on a glass slide with clear-drying glue. In instances where the left wing was damaged, the right wing was used. Wing images were collected using an Olympus X-70 microscope with bright field light at 10x. Wing length was measured, using the digital images, from the alula to the distal end minus the fringe scales to the nearest hundredth millimeter (ImageJ)^74^. Insect wing length measurements allow for a rapid estimate of body size due to strong correlations between wing length and indicators of adult size (adult dry weight^75^, pupal mass^76^).

### Statistical analysis

All statistical analysis was conducted in R v3.3.3^77^. To evaluate the significance of among-population differences on survival to adulthood (eclosion), proportional tests were conducted with two-way (Chi square) or three-way interactions (Cochran-Mantel-Haenszel)^77^. Survivorships (proportions surviving to eclosion) were compared among temperatures and states using a logistic regression^78^ with a binomial family and a logit link function with temperature and state as fixed effects. Genetic variation and phenotypic plasticity were assessed with ANOVA^78^, using temperature and state as fixed effects with maternal family as a random effect. Generalized linear mixed models (GLMM), using Penalized Quasi-Likelihood, of family means were created to assess the influence of temperature and state on the life history traits^79^. Sex was not a significant factor and was dropped from the final models. Survival analyses were performed for each state across the three temperatures using Kaplan-Meier survival analysis^80,81^. Differences between state and temperature results were compared using a Cox Regression^80^.

In order to test for the presence of a Bergmann’s cline, linear regression models were constructed to compare wing lengths of field-collected specimens over increasing latitude. To evaluate whether there was a genetic basis to this relationship, the same models were run on laboratory-reared specimens (means across states and temperature treatments).

## Supplementary information

Supplementary Information

Supplementary Dataset

## Data Availability

All relevant data are within the paper and its Supporting Information Files.
