# Supplementary material for: Regional variation in life history traits and plastic responses to temperature of the major malaria vector Nyssorhynchus darlingi in Brazil
Source: Sci Rep. 2019 Mar 29;9:5356. doi: 10.1038/s41598-019-41651-x (PMC6441093; doi:10.1038/s41598-019-41651-x)
Supplement: Supplementary file 1 — Supplementary Information [file 41598_2019_41651_MOESM1_ESM.pdf]

Regional variation in life history traits and plastic responses to temperature of the major malaria vector *Nyssorhynchus darlingi* in Brazil

V.M. Chu[1,2]*, M. A. M. Sallum[3], T. E. Moore[4], W. Lainhart[1,2,5], C.D. Schlichting[4] and J.E. Conn[1],[2]*

[1]Department of Biomedical Sciences, School of Public Health, University at Albany (State University of New York), Albany, NY, USA, [2] Wadsworth Center, New York State Department of Health, Albany, NY, USA, [3] Departamento de Epidemiologia, Faculdade de Saúde Pública, Universidade de São Paulo, SP, Brazil, [4] Department of Ecology and Evolutionary Biology, University of Connecticut, Storrs, CT, USA, [5] Current: Department of Pathology, University of Arizona College of Medicine, Tucson, AZ, USA, * co-corresponding authors

Supplementary- Methods

Climate variables were taken from WorldClim[82] with 30 second resolution from data spanning 1960-1990 and the NASA GISS-E2-R climate projection model under CMIP5 RCP85 forecast for 2070. The 2070 RCP85 climate data is for a high emission scenario, used in predictive modeling of spread of *Ny. darlingi* and *P. falciparum*[10]. Past and future bioclimate variables were aggregated for the same geographic coordinates to compare the climates with a principal component analysis. We used 19 bioclimate variables (Supplementary Table S5) drawn from 100 random points within the 4 states (Figure 1). Multiple measures of temperature and, to a slightly lesser extent, precipitation, are anticipated to change over the next 50 years in our study areas). The PCA was implemented using FactoMineR[86]and visualized using ggplot2[87].

Supplementary- Tables

All specimens (n=3430)			
**Effect**	**X^2**	**df**	**p-value**
Temperature	165.79	2	< 2.2e-16
State	256.19	3	< 2.2e-16
Temperature x State	10.824	6	0.09398

Supplementary Table S1. Proportional test results of temperature, state and their interaction on the number of surviving adults in total.

State	*Temperature* 20	24	28
Amazonas	0.91 (332, 31)	0.93 (333, 27)	0.87 (309, 48)
Rondônia	0.84 (373, 69)	0.85 (381, 65)	0.68 (302, 144)
Tocantins	0.69 (124, 55)	0.69 (119, 54)	0.53 (95, 85)
Rio de Janeiro	0.81 (129, 31)	0.77 (123, 36)	0.2 (33, 132)

Supplementary Table S2. Proportion of mosquitoes that survived to adulthood by state and temperature, with counts of dead adults and juveniles, respectively, in parentheses.

Fixed Effects	Coefficient	Std.Error	z-value	p-value
(Amazonas-20°C)	2.371	0.188	12.627	**1.51E-36**
Temperature- 24°C	0.141	0.274	0.514	6.07E-01
Temperature- 28°C	-0.509	0.244	-2.09	**3.67E-02**
Rondônia State	-0.684	0.229	-2.986	**2.83E-03**
Tocantins State	-1.558	0.248	-6.283	**3.33E-10**
Rio de Janeiro State	-0.945	0.274	-3.446	**5.70E-04**
Rondônia state- 24°C	-0.06	0.332	-0.181	8.56E-01
Rondônia state- 28°C	-0.438	0.295	-1.486	1.37E-01
Tocantins state- 24°C	-0.164	0.358	-0.457	6.47E-01
Tocantins state- 28°C	-0.193	0.328	-0.587	5.57E-01
Rio de Janeiro state- 24°C	-0.338	0.389	-0.87	3.84E-01
Rio de Janeiro state- 28°C	-2.303	0.37	-6.217	**5.06E-10**

Supplementary Table S3. Analysis of proportion of mosquitoes surviving to adulthood by state and temperature using a logistic regression with a binomial family and a logit link function with temperature and state and their interaction as fixed effects. Intercept is log odds of survival at 20°C for Amazonas state.

State	Temperature	Coefficient	Std.Error	z-value	p-value
Amazonas	20°C	-2.371	0.188	-12.626	**1.52E-36**
	24°C	-0.141	0.274	-0.514	6.07E-01
	28°C	0.509	0.244	2.09	**3.67E-02**
Rondônia	20°C	-1.687	0.131	-12.877	**6.04E-38**
	24°C	-0.081	0.188	-0.432	6.66E-01
	28°C	0.947	0.166	5.717	**1.08E-08**
Tocantins	20°C	-0.813	0.162	-5.018	**5.22E-07**
	24°C	0.023	0.231	0.099	9.21E-01
	28°C	0.702	0.22	3.185	**1.45E-03**
Rio de Janeiro	20°C	-1.426	0.2	-7.128	**1.02E-12**
	24°C	0.197	0.276	0.716	**4.74E-01**
	28°C	2.812	0.279	10.076	**7.03E-24**

Supplementary Table S4. Analysis of proportion of mosquitoes surviving to adulthood by state and temperature. Separate logistic regressions with a binomial family and a logit link function with temperature as a fixed effect. Intercepts are log odds of survival at 20°C.

Bioclimatic Variables			
Variable	Description	Coordinates	
		PC1	PC2
BIO1	Annual Mean temperature	-0.019	0.025
BIO2	Mean Diurnal Range (Mean of monthly (max temp - min temp))	-0.001	0.019
BIO3	Isothermality (2/7) (* 100)	-0.009	0.003
BIO4	Temperature Seasonality (standard deviation *100)	0.811	-0.548
BIO5	Max Temperature of Warmest Month	-0.011	0.031
BIO6	Min Temperature of Coldest Month	-0.030	0.017
BIO7	Temperature Annual Range (5-6)	0.018	0.015
BIO8	Mean Temperature of Wettest Quarter	-0.008	0.017
BIO9	Mean Temperature of Driest Quarter	-0.032	0.031
BIO10	Mean Temperature of Warmest Quarter	-0.008	0.018
BIO11	Mean Temperature of Coldest Quarter	-0.029	0.033
BIO12	Annual Precipitation	-0.514	-0.722
BIO13	Precipitation of Wettest Month	-0.049	-0.067
BIO14	Precipitation of Driest Month	-0.020	-0.058
BIO15	Precipitation Seasonality (Coefficient of Variation)	0.005	0.021
BIO16	Precipitation of Wettest Quarter	-0.162	-0.175
BIO17	Precipitation of Driest Quarter	-0.070	-0.192
BIO18	Precipitation of Warmest Quarter	0.014	-0.262
BIO19	Precipitation of Coldest Quarter	-0.199	-0.170

Supplementary Table S5. Bioclimatic variable description with corresponding coordinates. All bioclimatic variables from WorldClim[82].

Larvae development time (days)	Coefficient	Std.Error	d.f.	t-value	p-value
(Amazonas-20°C)	2.91	0.01	2566	199.44	**<.005**
Temperature- 24°C	-0.15	0.01	2566	-15.34	**<.005**
Temperature- 28°C	-0.29	0.01	2566	-26.78	**<.005**
Rondônia State	0.13	0.02	3	6.41	**0.01**
Tocantins State	0.16	0.02	3	7.00	**0.01**
Rio de Janeiro State	0.25	0.03	3	10.01	**<.005**
Rondônia state- 24°C	-0.05	0.01	2566	-3.91	**<.005**
Rondônia state- 28°C	0.00	0.01	2566	-0.18	0.86
Tocantins state- 24°C	0.05	0.02	2566	2.89	**<.005**
Tocantins state- 28°C	0.10	0.02	2566	5.21	**<.005**
Rio de Janeiro state- 24°C	-0.05	0.02	2566	-3.12	**<.005**
Rio de Janeiro state- 28°C	-0.05	0.03	2566	-1.91	0.06

Supplementary Table S6. GLMM results showing the effects of population, rearing temperature, and their interaction on larval development. Random effects: Locality and Family nested in Locality. *d.f.*-degrees of freedom

Pupal development time (days)	Coefficient	Std.Error	d.f.	t-value	p-value
(Intercept)	1.06	0.01	2566	132.61	**<.005**
Temperature- 24°C	-0.41	0.01	2566	-28.61	**<.005**
Temperature- 28°C	-0.56	0.02	2566	-33.78	**<.005**
Rondônia State	0.05	0.01	3	4.78	**1.74E-02**
Tocantins State	-0.12	0.02	3	-7.39	**5.10E-03**
Rio de Janeiro State	0.10	0.01	3	7.40	**5.10E-03**
Rondônia state- 24°C	-0.04	0.02	2566	-2.23	**2.58E-02**
Rondônia state- 28°C	-0.25	0.03	2566	-9.78	**<.005**
Tocantins state- 24°C	0.09	0.03	2566	3.01	**2.60E-03**
Tocantins state- 28°C	-0.24	0.04	2566	-5.48	**<.005**
Rio de Janeiro state- 24°C	-0.06	0.03	2566	-2.12	**3.38E-02**
Rio de Janeiro state- 28°C	-0.36	0.06	2566	-5.97	**<.005**

Supplementary Table S7. GLMM results showing the effects of population, rearing temperature, and their interaction on pupal development. Random effects: Locality and Family nested in Locality. *d.f.*-degrees of freedom

**Emergence time (days)**	**Coefficient**	**Std.Error**	**d.f.**	**t-value**	**p-value**
(Intercept)	3.05	0.01	2566	237.30	**<.005**
Temperature- 24°C	-0.18	0.01	2566	-20.77	**<.005**
Temperature- 28°C	-0.33	0.01	2566	-33.07	**<.005**
Rondônia State	0.12	0.02	3	6.56	**6.90E-03**
Tocantins State	0.12	0.02	3	6.01	**8.50E-03**
Rio de Janeiro State	0.23	0.02	3	10.47	**1.90E-03**
Rondônia state- 24°C	-0.05	0.01	2566	-4.10	**<.005**
Rondônia state- 28°C	-0.02	0.01	2566	-1.68	**9.38E-02**
Tocantins state- 24°C	0.06	0.02	2566	3.84	**1.00E-03**
Tocantins state- 28°C	0.08	0.02	2566	4.64	**<.005**
Rio de Janeiro state- 24°C	-0.05	0.01	2566	-3.22	**1.30E-03**
Rio de Janeiro state- 28°C	-0.07	0.02	2566	-2.91	**3.60E-03**

Supplementary Table S8. GLMM results showing the effects of population, rearing temperature, and their interaction on time to emergence. Random effects: Locality and Family nested in Locality. *d.f.*-degrees of freedom

**Adult longevity (days)**	**Coefficient**	**Std.Error**	**d.f.**	**t-value**	**p-value**
(Amazonas-20°C)	1.55	0.02	2566	92.89	**<.005**
Temperature- 24°C	-0.49	0.02	2566	-28.36	**<.005**
Temperature- 28°C	-0.86	0.02	2566	-42.66	**<.005**
Rondônia State	-0.14	0.02	3	-6.19	**0.01**
Tocantins State	-0.30	0.03	3	-9.18	**<.005**
Rio de Janeiro State	0.00	0.03	3	0.08	0.94
Rondônia state- 24°C	0.14	0.02	2566	5.89	**<.005**
Rondônia state- 28°C	-0.01	0.03	2566	-0.22	0.83
Tocantins state- 24°C	0.09	0.04	2566	2.40	**0.02**
Tocantins state- 28°C	0.22	0.04	2566	5.16	**<.005**
Rio de Janeiro state- 24°C	-0.10	0.03	2566	-2.94	**<.005**
Rio de Janeiro state- 28°C	-0.04	0.06	2566	-0.74	0.46

Supplementary Table S9. GLMM results showing the effects of population, rearing temperature, and their interaction on adult longevity. Random effects: Locality and Family nested in Locality. *d.f.*-degrees of freedom

Wing length (mm)	Coefficient	Std.Error	d.f.	t-value	p-value
(Amazonas-20°C)	1.02	0.00	2493	256.23	**<.005**
Temperature- 24°C	-0.06	0.00	2493	-16.98	**<.005**
Temperature- 28°C	-0.09	0.00	2493	-23.62	**<.005**
Rondônia State	0.01	0.01	3	2.51	**0.09**
Tocantins State	0.01	0.01	3	1.96	0.14
Rio de Janeiro State	0.08	0.01	3	11.66	**<.005**
Rondônia state- 24°C	0.00	0.00	2493	0.65	0.52
Rondônia state- 28°C	-0.02	0.01	2493	-4.84	**<.005**
Tocantins state- 24°C	0.03	0.01	2493	4.91	**<.005**
Tocantins state- 28°C	0.01	0.01	2493	0.98	0.32
Rio de Janeiro state-24°C	-0.02	0.01	2493	-2.71	**0.01**
Rio de Janeiro state-28°C	-0.04	0.01	2493	-4.19	**<.005**

Supplementary Table S10. GLMM results showing the effects of population, rearing temperature, and their interaction on wing length. Random effects: Locality and Family nested in Locality. *d.f.*-degrees of freedom

**Biome**	Amazon				Cerrado		Mata Atlântica
**State**	Amazonas		Rondônia		Tocantins		Rio de Janeiro
**Collection Month**	October		July		March		May
**Months high precipitation***	January-April		December-March		November-February		October-January
**Locality**	ARS	APR	RPV	RMO	TLC	TPN	SJU
**Latitude**	-2.864	-3.028	-8.742	-9.223	-10.7	-10.796	-22.611
**# Females collected**	20	53	95	45	34	14	15
**# Females allowed to lay eggs**	20	50	50	45	34	14	15
**# Females laid eggs in lab**	12	39	40	33	9	6	12
**# Families used in life history study**	10	15	15	15	8	4	11

Supplementary Table S11. Field collected female data. All collections occurred in 2016.
*Precipitation data from monthly averages collected from 1961-1990[84-85].

Supplementary- Figures

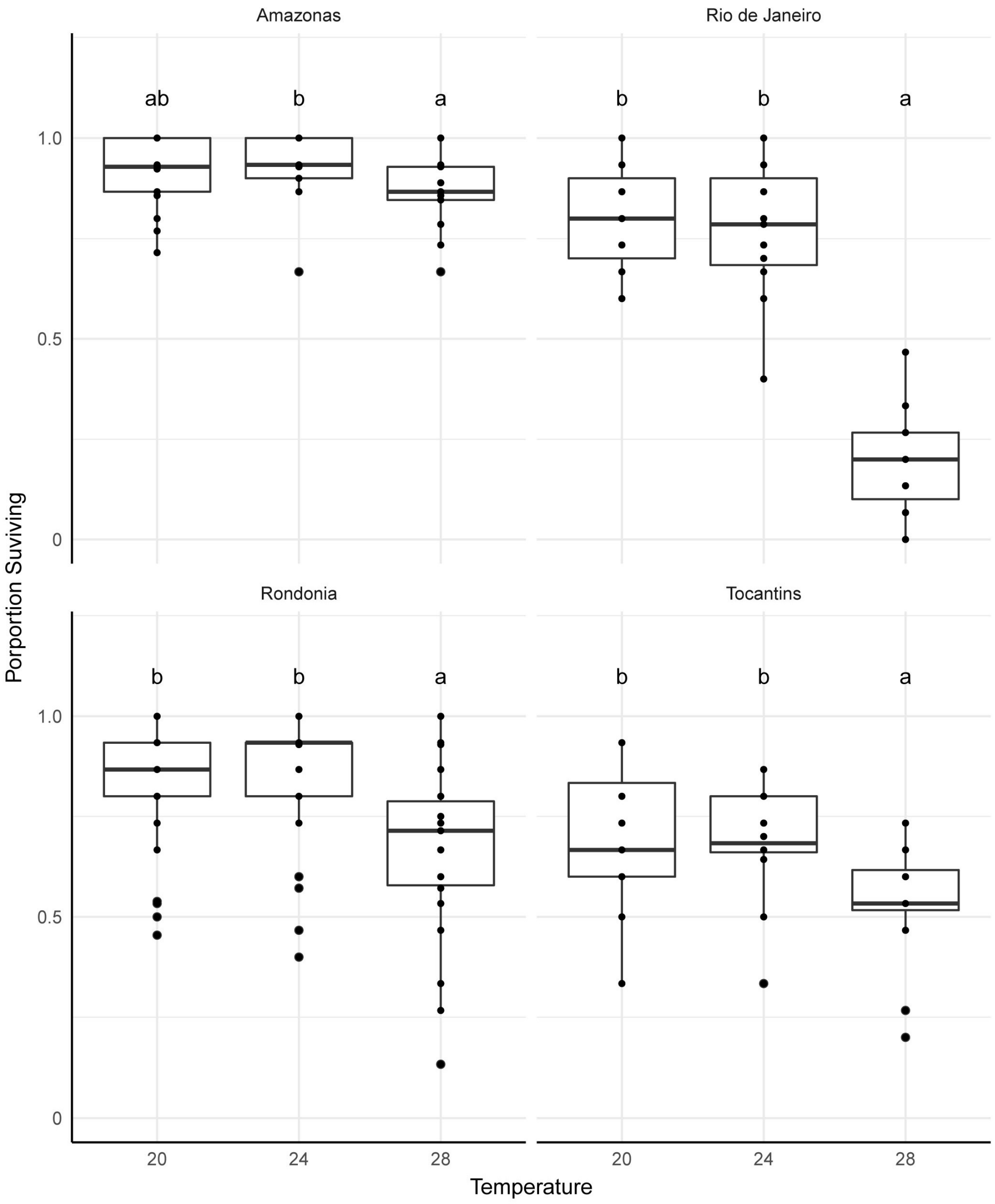

Supplementary Figure S1. Boxplots of survival to eclosion as adults, by state and temperature. Boxplots show means and interquartile ranges. Each point is the mean of a maternal family. Different letters within a state indicate significant differences in proportion surviving among temperatures (see Supplementary Table S2).

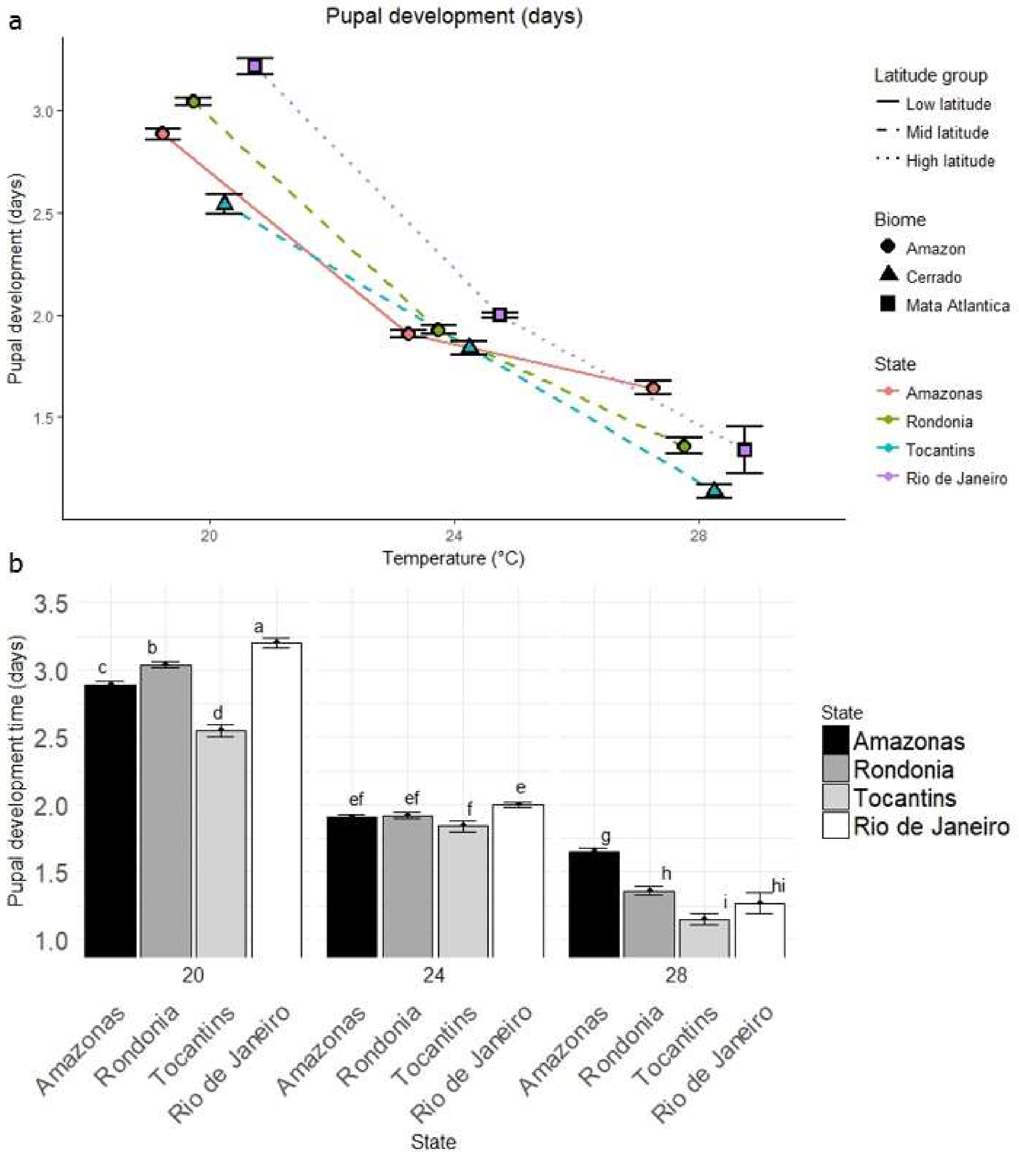

Supplementary Figure S2. Average duration of pupal development times (days) with standard error by state (a). Average pupal development times (days) (b) with standard error. Letters above bars are derived from Tukey HSD after GLMM and indicate significantly different groups (*p*-value <0.05).

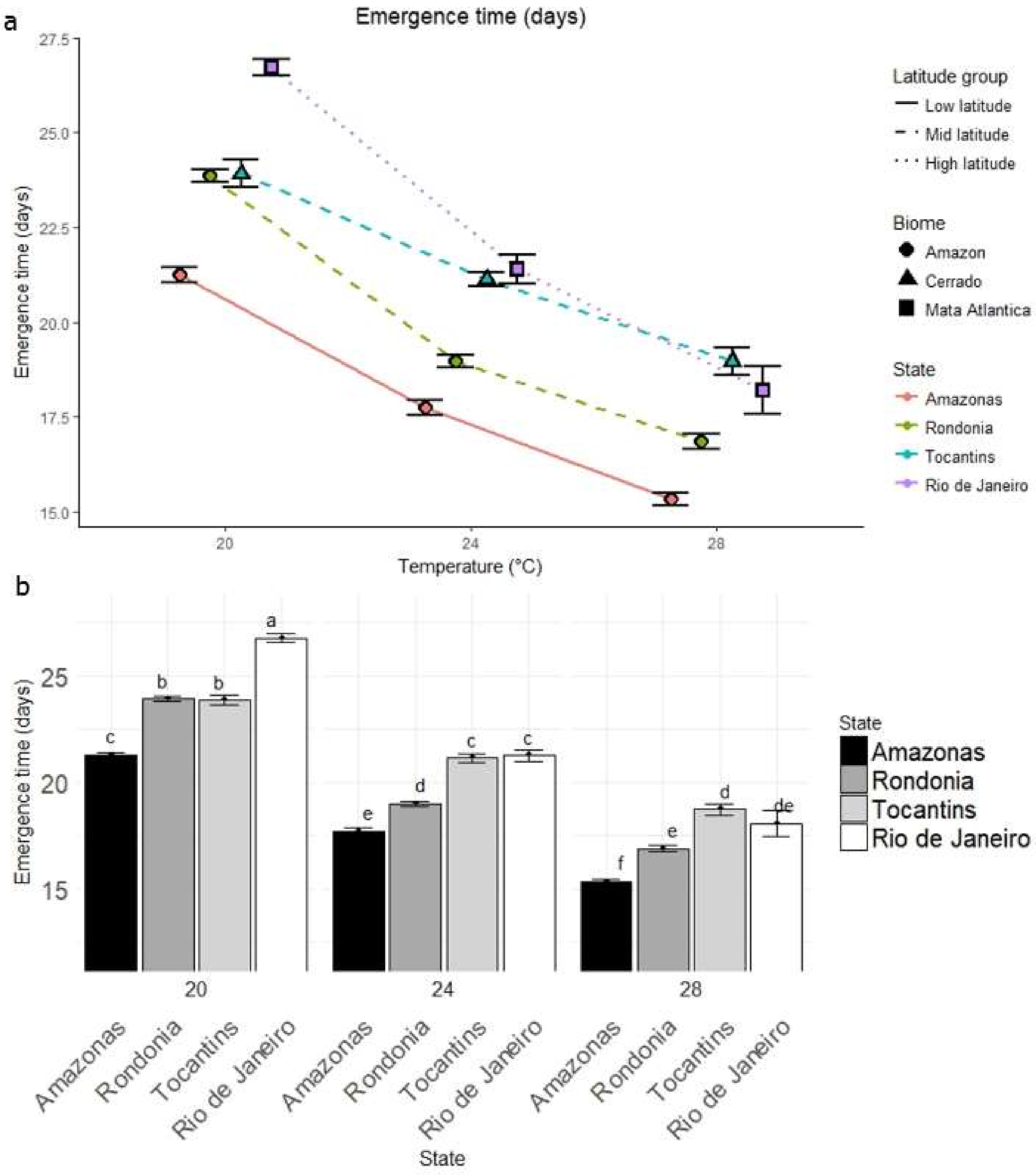

Supplementary Figure S3. Average time to adult emergence (days) with standard error by state (a). Average pupal development times (days) (b) with standard error is graphed. Letters above bars derived from Tukey HSD of data after GLMM and indicate significantly different groups (*p*-value <0.05).

Supplementary Figure S4. Average duration of larvae development (days, **a**), average adult life (days, **b**), and average wing length (mm, **c**) with standard error bars. Letters above bars derived from Tukey HSD after GLMM, with different letters indicate significantly different groups (p-value <0.05).

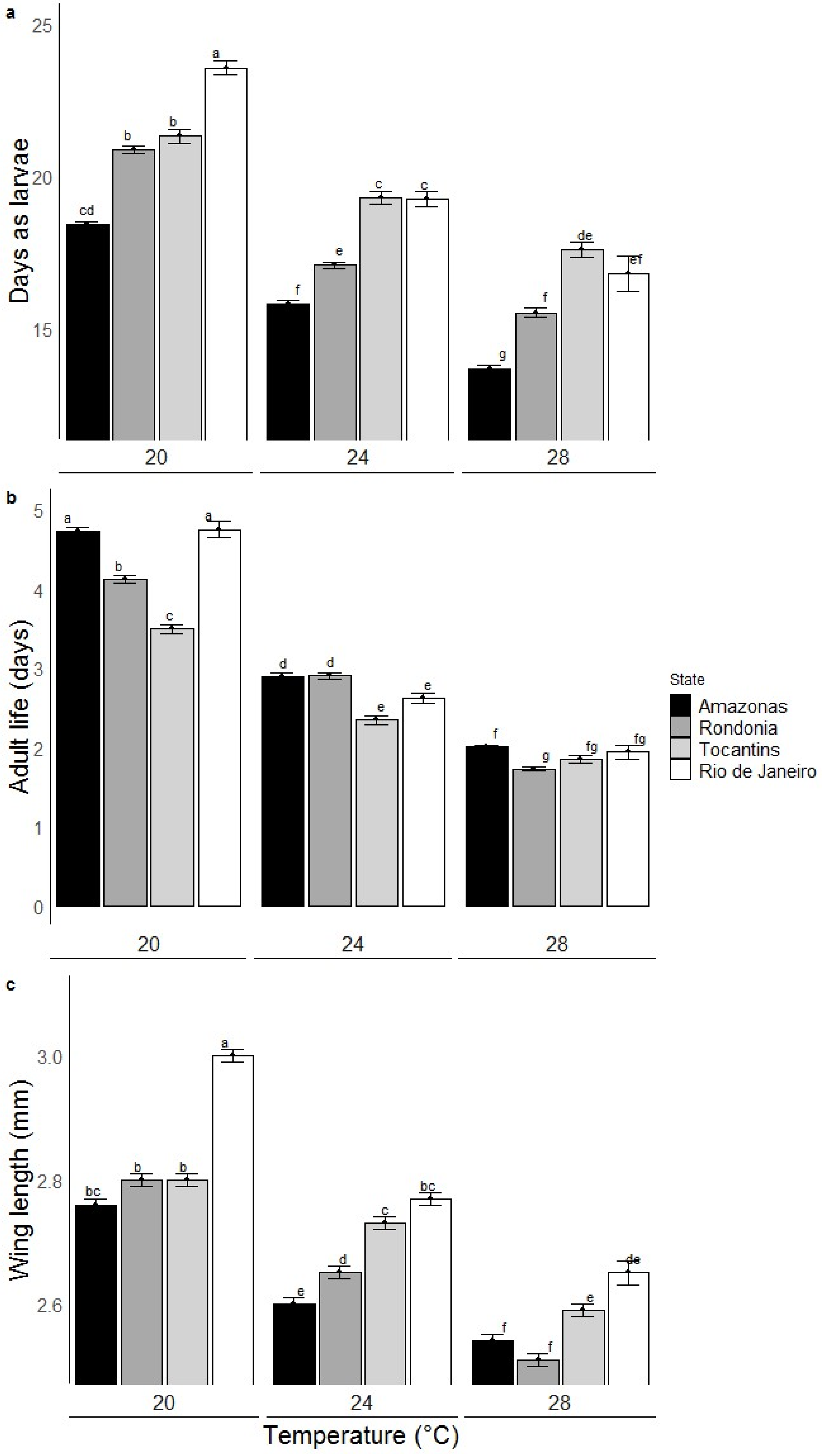

References

86. Sebastien Le, Julie Josse, F. H. FactoMineR: An R Package for Multivariate Analysis. *J. Stat. Softw.* **25,** 1–18 (2008).

87. Wickham, H. *ggplot2: Elegant Graphics for Data Analysis*. (Springer-Verlag New York, 2009).
